# Sesquiterpenes of the ectomycorrhizal fungus *Pisolithus microcarpus* alter root growth and promote host colonization

**DOI:** 10.1007/s00572-024-01137-9

**Published:** 2024-03-05

**Authors:** Jonathan M. Plett, Dominika Wojtalewicz, Krista L. Plett, Sabrina Collin, Annegret Kohler, Christophe Jacob, Francis Martin

**Affiliations:** 1 Hawkesbury Institute for the Environment, Western Sydney University, Richmond, NSW 2753 Australia; 2 Elizabeth Macarthur Agricultural Institute, NSW Department of Primary Industries, Menangle, NSW 2568 Australia; 3 Université de Lorraine, CNRS, IMoPA F-54000 Nancy, France; 4 Université de Lorraine, INRAE, UMR Interactions Arbres-Microorganismes, Centre INRAE Grand Est-Nancy, 54280 Champenoux, France

**Keywords:** Terpene synthase, Mycorrhiza, Symbiosis, Plant-microbe interaction, Root, Fungus

## Abstract

Trees form symbioses with ectomycorrhizal (ECM) fungi, maintained in part through mutual benefit to both organisms. Our understanding of the signaling events leading to the successful interaction between the two partners requires further study. This is especially true for understanding the role of volatile signals produced by ECM fungi. Terpenoids are a predominant class of volatiles produced by ECM fungi. While several ECM genomes are enriched in the enzymes responsible for the production of these volatiles (i.e., terpene synthases (TPSs)) when compared to other fungi, we have limited understanding of the biochemical products associated with each enzyme and the physiological impact of specific terpenes on plant growth. Using a combination of phylogenetic analyses, RNA sequencing, and functional characterization of five TPSs from two distantly related ECM fungi (*Laccaria bicolor* and *Pisolithus microcarpus*), we investigated the role of these secondary metabolites during the establishment of symbiosis. We found that despite phylogenetic divergence, these TPSs produced very similar terpene profiles. We focused on the role of *P. microcarpus* terpenes and found that the fungus expressed a diverse array of mono-, di-, and sesquiterpenes prior to contact with the host. However, these metabolites were repressed following physical contact with the host *Eucalyptus grandis*. Exposure of *E. grandis* to heterologously produced terpenes (enriched primarily in $\gamma$-cadinene) led to a reduction in the root growth rate and an increase in *P. microcarpus*–colonized root tips. These results support a very early putative role of fungal-produced terpenes in the establishment of symbiosis between mycorrhizal fungi and their hosts.

**Supplementary Information:**

The online version contains supplementary material available at 10.1007/s00572-024-01137-9.

## Introduction

For all living organisms, secondary metabolites are critical to intra- and inter-cellular communication. These metabolites fall into four predominant chemical classes, of which terpenoids are the largest in plants and fungi. Composed of isoprenoid subunits (i.e., C5 hemiterpenes), this class of secondary metabolites can be linear or cyclic, can have varying levels of saturation, and may be soluble or volatile (Chen et al. 2021; Zhou and Pichersky 2020). The synthesis of a terpenoid typically begins with a single terpene synthase (TPS) enzyme that results in cyclization of the carbon-rich backbone, after which a range of different enzymes can functionalize the compound in a unique manner. Owing to the flexibility of these biosynthetic pathways, more than 400 structural families and > 80,000 individual terpenoids have been described (Helfrich et al. 2019). While the breadth of these compounds has impeded a broader understanding of how they function, great strides have been made in their role in plant and fungal development and in the control of plant-microbe interactions.

The most widely studied terpenoids in plants are hormones, including gibberellin (GA), abscisic acid (ABA), and cytokinins (Chen et al. 2020a, b; Cortleven et al. 2019; Hedden 2020). These factors play a role in plant development, including seed germination, growth, fruit ripening, and the loss of senescing plant tissues. They can also play an indirect role in plant-microbe interactions with elevated levels of ABA or GA impeding the ability of pathogenic fungi to colonize plant tissues (Bharath et al. 2021; Buhrow et al. 2021; Ton and Mauch-Mani 2004; Toner et al. 2021). Conversely, ABA promotes root colonization by arbuscular mycorrhizal (AM) fungi (Chapentier et al. 2014; Lou et al. 2021) and increases the fungal biomass in plant roots in ectomycorrhizal (ECM) fungal–colonized root tips (Hill et al. 2022). Other terpenoids are also involved in direct defense against microbes or insects. Cotton cultivars resistant to the pathogen *Verticillium dahliae* have been characterized by higher and faster production of a number of terpenoids including $\delta$-cadinene (Bianchini et al. 1999), whereas cucurbits release triterpenoids into the rhizosphere that act to promote a beneficial microbiome (Zhong et al. 2022). Elevated production of linalool in strawberries during infection with *Botrytis cinerea* inhibits the production of fungal ergosterol, compromising membrane integrity and leading to collapse of the mitochondrial membrane (Xu et al. 2019). The process of fungal colonization of plants can also reduce terpene production in the host. During the colonization of plant tissues by mutualistic fungi such as the ECM fungus *Laccaria bicolor,* the expression of host terpene synthases must be attenuated to enable symbiosis (Marqués-Gálvez et al. 2022). Inoculation of cork oak with *Raffaelea montetyi*, *R. quercina*, and *Ceratocystiopsis* sp. nov., four fungi farmed by ambrosia beetles during their establishment within the host plant, leads to a reduction in the host biosynthesis of monoterpenes, possibly as a means of controlling plant immunity (Nones et al. 2022). Fungal colonization can also systemically affect terpene synthesis (Sarkar and Sadhukhan 2023). For example, arbuscular mycorrhizal colonization of roots leads to a doubling of terpene production in tomato leaves, rendering them more resistant to armyworms, a caterpillar pest (Shrivastava et al. 2015).

Fungi can also synthesize a range of terpenes, which, like plant terpenes, have been studied primarily in the context of defense. For example, the plant endophyte *Phomopsis cassiae* produces five different compounds within the cadinane class of sesquiterpenes, of which 3,11,12-trihydroxycadalene acts as a biocontrol compound against two species of *Cladosporium* (Silva et al. 2006). Fungal terpenes may also promote the colonization of host plant tissues. Terpenes released by the ECM fungus *L. bicolor,* the most abundant being α-muurolene, γ-selinene, and δ-cadinene, were found to promote host lateral rooting by over 300% (Ditengou et al. 2015). Interestingly, the authors of this study found that this increase was not tied to the most abundant terpenes, but to the sesquiterpene thujopsene. Volatiles enriched in terpenes of the non-mycorrhizal *Trichoderma viride* have been shown to improve plant growth and increase lateral rooting (Hung et al. 2013). Pine-associated *Tricholoma vaccinum* was found to produce 20 sesquiterpenes in axenic cultures, 2 of which increased during host colonization (Δ^6^-protoilludene and β-barbatene) and an unidentified oxygenated sesquiterpene (m/z 218.18) that was repressed (Abdulsalam et al. 2021; Ezediokpu et al. 2022). Not all ECM fungi can produce complex volatile terpenoids, for example, the ascomycete ECM fungus *Cenococcum geophilum, which* is only able to produce monoterpenes that are repressed in the presence of plant roots (Ditengou et al. 2015).

Within mutualistic ECM fungi, the majority of our knowledge concerning potential roles for terpenes in communicating with plant hosts comes from transcriptomic and comparative genomic studies (Chot et al. 2023; Lebreton et al. 2022; Nosenko et al. 2023; Ruytinx et al. 2021). For example, terpene synthesis in the ECM fungus *Piloderma croceum* was found to be significantly regulated (Liao et al. 2014). Comparative genomics within the *Suillus* genus found that terpene synthase profiles varied depending on the host specificity of the species and that *Suillus* as a whole had a significantly higher number of terpene synthase secondary metabolite clusters than other ECM fungi (Lofgren et al. 2021). However, in both cases, the functional implications of the different terpene profiles are not known. Therefore, further characterization of terpene synthase products in symbiotic fungi is required, and the functional role of these compounds is tested. To address this, we sought to functionally identify the products of terpene synthases from two distantly related ECM fungi, *L. bicolor* and *Pisolithus microcarpus.* We further investigated the role of *P. microcarpus* terpenes during symbiosis with *Eucalyptus grandis.* From the work performed with *L. bicolor* and *T. vaccinum,* we hypothesized that terpene synthase gene expression and the production of terpenes in *P. microcarpus* would increase during the early stages of colonization. Based on previous work on the *L. bicolor:Populus* model system, we hypothesized that *P. microcarpus* terpenes would induce lateral rooting of the host and improve colonization. Answers to these hypotheses will add to our understanding of the dynamics of early mycorrhizal signaling leading to symbiosis.

## Methods

### Phylogenetic tree reconstruction of plant and fungal TPSs

We downloaded 200 putative TPSs from published basidiomycete genomes in MycoCosm, spanning saprotrophic, pathogenic, and mutualistic fungi. These included *Agaricus bisporus* var. burnettii JB137-S8 (Morin et al. 2012), *Ceriporiopsis* (*Gelatoporia*) *subvermispora* B (Fernandez-Fueyo et al. 2012), *Coniophora puteana* (Floudas et al. 2012), *Coprinopsis cinerea* (Stajich et al. 2010), *Ganoderma* sp. 10597 SS1 (Binder et al. 2013), *Hypholoma sublateritium* (Kohler et al. 2015), *Jaapia argillaceae* (Riley et al. 2014), *Paxillus involutus* ATCC 200175 (Kohler et al. 2015), *Phanerochaete chrysosporium* RP-78 (Ohm et al. 2014), *Pleurotus ostreatus* PC15 (Riley et al. 2014), *Serpula lacrymans* S7.3 (Eastwood et al. 2011), *Omphalotus olearius* (Wawrzyn et al. 2012), *P. microcarpus* (Kohler et al. 2015), *and Laccaria bicolor* (Martin et al. 2008). For plant TPSs, we downloaded all putative synthases from *Arabidopsis thaliana* (Arabidopsis Genome Initiative 2000), *Gossypium hirsutum* (Chen et al. 2020a, b), and *E. grandis* (Myburg et al. 2014). Phylogenetic analysis was conducted using the Phylogeny.fr online tool (Dereeper et al. 2008). All protein sequences were aligned using MUSCLE 3.8.31, and poorly aligned positions and divergent regions were eliminated using Gblocks version 0.91b. The phylogenetic tree was constructed using the maximum likelihood method (PhyML3.1/3.0 aLRT) and drawn using TreeDyn 198.3. Putative terpene products of plant TPS phylogenetic groups were applied based on either Phytozome annotations (https://phytozome-next.jgi.doe.gov/; last accessed April 2023) or functional annotation by Külheim and colleagues (2015).

### TPS cloning and expression

Three putative TPS-coding proteins from *L. bicolor* (Lb_312850, Lb_393120, and Lb_394925) and two from *P. microcarpus* (Pmic_85821 and Pmic_685262) were cloned from cDNA generated from RNA extracted from free-living mycelia (FLM). Coding sequences were amplified using KAPA HiFi HotStart ready mix according to the manufacturer’s instructions (Roche), gel purified using the Wizard SV Gel cleanup kit (Promega), and cloned into pET28a between *Nde*I and *Sal*I restriction sites using InFusion according to the manufacturer’s instructions (Scientifix Pty Ltd.). The resulting plasmid or an empty vector control was used to transform the Rosetta2 (DE3) pLysS strain of *E. coli* (Novagen). The expression of the recombinant proteins or empty vector control was performed at 37°C in Lysogeny broth medium until the cell culture reached an OD_600_ of 0.6. Recombinant protein expression was induced by the addition of 0.1 mM isopropyl β-D-1-thiogalactopyranoside (IPTG), and the cells were grown for 24 h at room temperature. The terpene profiles were analyzed using solid-phase microextraction (SPME), as described below. We performed terpene expression with and without the addition of geranyl pyrophosphate and farnesyl pyrophosphate, but we observed no difference in terpenoid profiles. Therefore, we only report the results obtained without the addition of these substrates.

### Microcosm establishment

For *P. microcarpus* colonization experiments, *E. grandis* seeds (Seedlot 20974; Commonwealth Scientific and Industrial Research Organization Tree Seed Center; Clayton, VIC Australia) were sterilized in 30% H_2_O_2_ (v/v) and germinated on 1% (w/v) water agar for 1 month in a controlled incubator at 25 °C with a 16-h light cycle. Following this, seedlings were transferred into half-strength modified Melin-Norkrans (1/2 MMN) media (1.9 mM (NH_4_)_2_PO_4_; 1.1 mM KH_2_PO_4_; 0.28 mM MgSO_4_ 7H_2_O; 5.5 mM glucose; 0.23 mM CaCl_2_; 0.22 mM NaCl; 0.008 mM ZnSO_4_; 0.002 mM thiamine; 0.033 mM citric acid; 0.018 mM Fe-EDTA in a 1.3% agar solution at pH 5.5) placed between two cellophane membranes to prevent growth into the media (Kleerview Covers by Fowlers Vacola Manufacturing Co. Ltd.) and grown for another month (22–30 °C night/day temperature; 16-h light cycle). Two weeks before contact with the plants, *P. microcarpus* isolate SI-14 (Plett et al. 2015) was propagated on cellophane-covered half-strength MMN plates and grown in the dark at 25 °C. Once the *E. grandis* seedlings were 2 months old and the fungal cultures were 2 weeks old, the plants were separated into one of three treatment categories: “axenic controls” consisted of seedlings transferred onto new half-strength MMN medium without any fungal inoculum; “pre-symbiosis seedlings” were placed in indirect contact with fungal mycelium by separating the two organisms by a permeable cellophane membrane for 24 h; and “physical contact” seedlings were placed into direct contact with the fungal mycelium, and then, individual biological replicates were destructively harvested at 24 h, 48 h, 1 week, and 2 weeks post-contact. Three biological replicates per time point were used for the gene expression experiments. All Petri dishes were closed with gas permeable Parafilm for the duration of the experiment.

To test the phenotypic impact that terpene volatiles would have on root growth and fungal colonization, at the beginning of the colonization period (i.e., “physical contact”), seedlings with or without fungal inoculum were transferred to cellophane-covered ½ MMN in one half of a two-compartment 90-mm Petri dish. The second half of the plate contained a 20-mm petri dish lid into which 500 *µ*L of IPTG-induced *E. coli* carrying either an empty vector (pET28a) or Pmic_685262-pET28a was added. The plates were sealed with Parafilm and placed in a growth chamber. The induced *E. coli* culture was replaced with fresh culture every 24 h for 2 weeks, after which root growth, lateral root appearance, and colonization were scored as previously described in Plett et al. (2015). The experiment was repeated twice with a minimum of 15 biological replicates per treatment. Terpene enrichment in the headspace of the microcosms was verified using SPME as described below.

### SPME and gas chromatography-mass spectrometry (GC-MS)

Volatile terpene profiles were analyzed using SPME (solid-phase microextraction; Suppleco) fibers chosen based on the manufacturers’ selection criteria and verified using in-house experiments to test the ability of the fibers to absorb volatile compound with molecular weight below 250 m/z. To that end, we prepared four identical 20-mL vials holding 10 mL of the induced *E. coli* Rosetta2 (DE3) pLysS expressing *P. microcarpus* protein 685262 and exposed each type of SPME fiber for 1 h in the headspace area and analyzed the trapped volatiles by GC-MS to identify the fiber chemistry that collected the highest diversity of sesquiterpenes and terpenoids. Based on these analyses, we used a SPME fiber coating of Carboxen/polydimethylsiloxane (85 *µ*m CAR/PDMS) in all future experiments for collecting headspace products. SPME fibers were conditioned before use as per the manufacturer’s recommendation and checked routinely to ensure no cross-contamination between samples by performing blank analysis between samples.

To collect terpenes enriched within the headspace of the microcosms, we inserted a SPME fiber through a small hole burnt on the side of each Petri dish at the sampling times listed in the manuscript. The fiber was exposed for 1 h of absorption time within this headspace, while the microcosms were maintained in the same growing conditions as described above. After this exposure time, each SPME fiber was inserted into fiber holder and loaded on a Gerstel Multipurpose Sampler. Employing this automated sampler system helped to ensure reproducibility of the injection and desorption process in the GC injection port, set up to 250 °C and splitless mode with a desorption time of 120 s. Chromatography analyses were performed on an Agilent 7890A Gas Chromatograph coupled with 5975C inert XL Mass Spectrometer with Triple Axis detector equipped with Agilent J&W GC column 60 m, 0.25 mm ID, and 0.25 *µ*m film thickness. The gas chromatograph oven temperature program was set up as follows: 50 °C for 2 min, 5 °C /min 50–200 °C, and 20 °C /min 200–250 °C at which point the injector was maintained at the highest temperature for 2 min. The mass spectrometer was set up to maintain ion source and quad temperature at 230 °C and 150 °C, respectively. The analyses were performed in scan mode, collecting all ions in the range 50–600 m/z and checking carefully the mass spectrum of all potential sesquiterpenes peaks. To confirm the identity of each detected peak, we used the digital mass spectral library-identification of essential oil components by gas chromatography/mass spectrometry, by Adams (2017). To ensure correctness of the identified peaks, we compared mass spectrum and values of AI (arithmetic index) on the Agilent J&W DB-5 GC column in reference to n-alkanes of detected peaks with the library data.

### Gene expression analysis

*P. microcarpus* and *E. grandis* gene expression of putative terpene synthases was performed on RNA sequencing dataset as reported in Hill and colleagues (2022). Briefly, microcosms were established as described above, and three biological replicates were taken at each of the time points. RNA was extracted using the ISOLATE II Plant miRNA kit (Bioline) and sequenced according to Illumina TruSeq Stranded mRNA HT sample preparation guidelines. mRNA, using a Poly-A library construction, sequencing was performed on the Illumina HiSeq sequencing platform following a 2 × 150 paired-end sequencing protocol. These raw RNA-seq reads were then evaluated for artifact sequences by kmer matching (kmer = 25), allowing one mismatch and detected artifact, trimmed from the 3′ end of the reads using BBDuk (https://sourceforge.net/projects/bbmap/). The filtered reads from each RNA-seq library were then aligned with the *E. grandis* (Myburg et al. 2014) and *P. microcarpus* genomes (Plett et al. 2023). For *E. grandis*, an “expressed” gene was defined as one whose normalized expression was greater than two fragments per kilobase of transcript per million mapped reads (FPKM), at any time point considered, while for *P. microcarpus*, this was greater than five FPKM. Significantly differentially expressed genes (DEGs) were identified using DEseq2 in R-Studio (Love et al. 2014). Relative Log2 fold change was calculated through pairwise comparison, whereby a significant DEG was deemed to have a fold change of greater than 2× and a *P* < 0.05 (adjusted for false discovery rate). Heat map figures were produced using HeatMapper (https://heatmapper.ca/).

## Results

### Ectomycorrhizal fungi encode terpene synthases catalyzing sesquiterpene synthesis

**Fig. 1:**
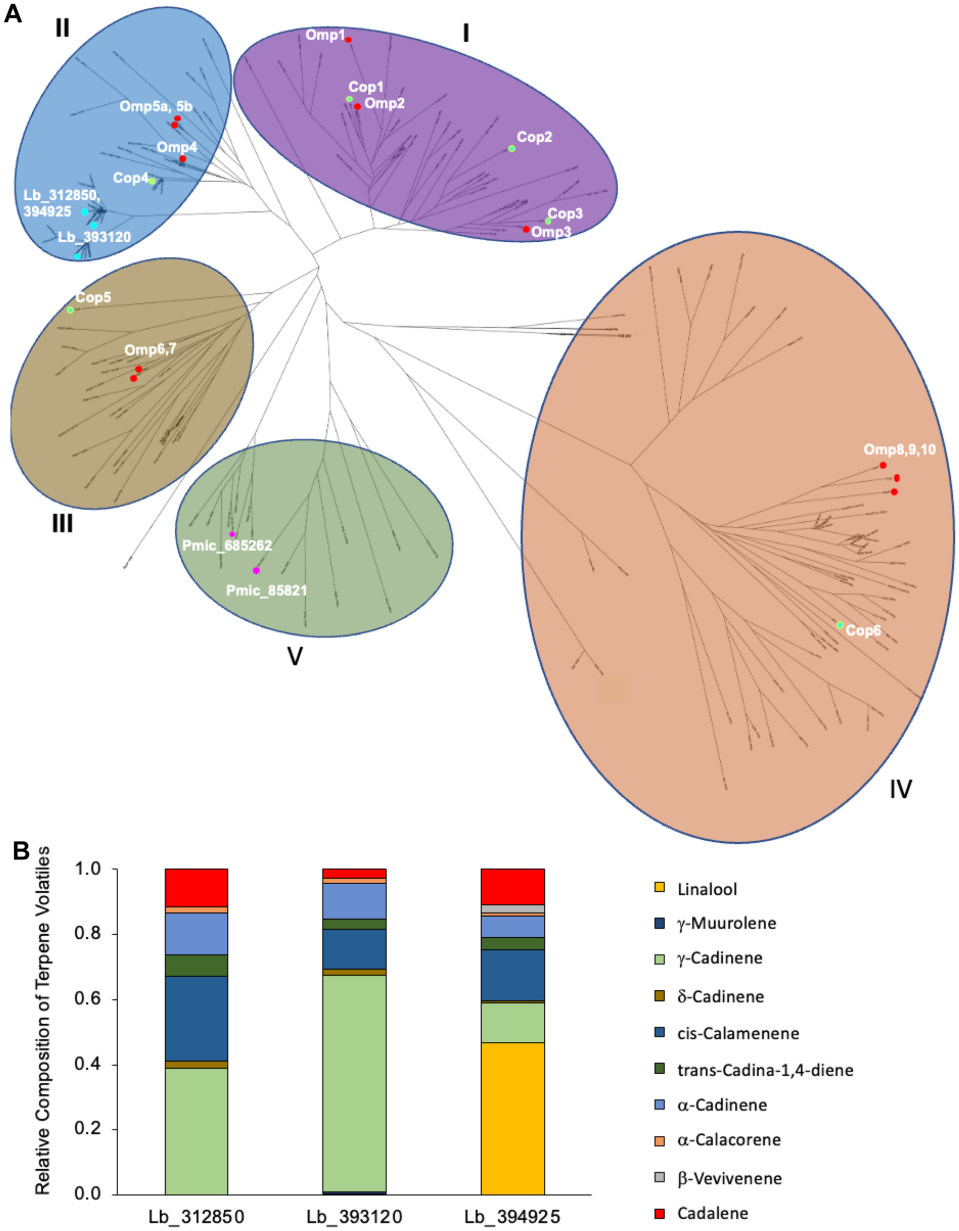
Terpene synthases of ectomycorrhizal fungi are found in multiple phylogenetic clades and produce a range of volatiles. **A** Unrooted maximum likelihood phylogram of TPS homologs identified in 14 Basidiomycota genomes with the five clades identified by Wawrzyn and colleagues (2012) highlighted in colors. Previously characterized TPSs from *C. cinereus* (Cop) and *O. olearius* (Omp) were labeled as were *L. bicolor* (Lb) and *P. microcarpus* (Pmic) proteins. Please consult the “Methods” section for tree construction parameters. **B** Volatile metabolites produced by liquid cultures of *E. coli* Rosetta2 (DE3) pLysS heterologously expressing either *L. bicolor* proteins Lb_312850, Lb_393120, and Lb_394925 or an empty vector control. The headspace above the induced cultures was sampled by solid-phase microextraction (SPME) and analyzed by gas chromatography-mass spectrometry (GC-MS). The identified sesquiterpene compounds that were not found in *E. coli* Rosetta2 (DE3) pLysS cultures containing an empty expression vector are reported here as percentages of the total identified sesquiterpenes using semi-quantitative analysis

Previous phylogenetic analyses of basidiomycete fungal genomes found that TPS-encoding genes ranged from 16 to 41 copies per genome and that these sequences fell into one of five clades (Wawrzyn et al. 2012). We sought to revisit this analysis given recent updated genomes now available for the two mutualistic ectomycorrhizal fungi *L. bicolor* and *P. microcarpus* (Miyauchi et al. 2020; Plett et al. 2023). In the former genome, after manual curation of genes annotated as containing a TPS domain to remove sequences without predicted start codons or that contained multiple nonsense mutations, *L. bicolor* was found to encode 44 putative TPSs. *P. microcarpus,* meanwhile, was found to encode 14 putative TPSs. The TPSs of basidiomycete fungi fell within five main clades (Fig. 1A). The majority of *L. bicolor* sequences fell within clade II, grouping with other enzymes with proven roles in 1,10 cyclization of *3R-*NPP, and clade IV, grouping with enzymes responsible for 1,6 or 1,7 cyclization of the same substrate (Wawrzyn et al. 2012). The TPS-coding sequences of *P. microcarpus* were found to spread more broadly throughout the different clades, including clade V, whose function is uncertain (Fig. 1A).

As several *L. bicolor* TPS-encoding genes have been previously identified to be induced during symbiosis with its plant host *Populus trichocarpa*, we sought to experimentally verify their ability to catalyze the synthesis of terpenes. The three *L. bicolor* genes most induced during symbiosis code for proteins Lb_312850, Lb_393120, and Lb_394925 (Martin et al. 2008), all of which fall within clade II and are predicted to encode $\delta$-cadinene synthases based on their similarity to previously characterized proteins within this clade (that is, Omp4, 5a/b; Cop4; Fig. 1A). When expressed individually in *E. coli,* these three proteins result in the production of a mixture of sesquiterpenes (Fig. 1B). *E. coli* expressing an empty expression vector was only found to produce indole, and therefore, we did not include this volatile in the analyses. While $\delta$-cadinene was detected as a product of all three synthases, it was one of the least abundant volatiles which accounted for 2% of the mono- and di-terpenoids detected in bacteria expressing *L. bicolor* proteins Lb_312850 and Lb_393120 and only 1% in the volatile terpenes produced when Lb_394925 was expressed. The major product of Lb_312850 and Lb_393120 was $\gamma$-cadinene (39% and 67%, respectively; Table 1) with *cis*-calamenene being the second-most abundant terpenoid (26% and 12%, respectively; Table 1). Despite showing high sequence similarity to the other two proteins (e.g., Lb_394925 is 84.7% similar to Lb_393120; Table 1), Lb_394925 produced a high concentration of linalool (47% of all detected volatile terpenoids) with *cis*-calamenene and $\gamma$-cadinene being secondary in abundance (16% and 12%, respectively; Table 1).

**Table 1:** Terpenoid profile of *E. coli* heterologously producing terpene synthases of either *L. bicolor* (Lb_) or *P. microcarpus* (Pmic_)

**Retention time**	**KI**	**Compound name**	**Category**	**Formula**	**CAS#**	**Percentage distribution of terpenes**				
						**Lb_312850**	**Lb_393120**	**Lb_394925**	**Pmic_85821**	**Pmic_685262**
11.91	979	$\beta$-Pinene	Monoterpene	C_10_H_16_	127-91-3	0.0	0.0	0.0	0.0	0.5
12.31	1000	meta-Mentha-1(7),8-diene	Monoterpene	C_10_H_16_	13837-95-1	0.0	0.0	0.0	0.0	10.6
14.05	1007	iso-Sylvestrene	Monoterpene	C_10_H_16_	499-03-6	0.0	0.0	0.0	0.0	0.3
14.46	1095	Linalool	Monoterpenoid	C_10_H_18_O	78-70-6	0.0	0.0	46.9	0.0	0.0
23.12	1430	$\beta$-Copaene <beta->	Sesquiterpene	C_15_H_24_	18252-44-3	0.0	0.0	0.0	0.0	0.6
25.00	1475	*trans*-cadina-1(6),4-diene	Sesquiterpene	C_15_H_24_	20085-11-4	0.0	0.2	0.0	0.4	0.2
25.23	1478	$\gamma$-Muurolene	Sesquiterpene	C_15_H_24_	30021-74-0	0.0	0.8	0.0	0.0	0.2
25.64	1505	$\alpha$-Cuprenene	Sesquiterpene	C_15_H_24_	29621-78-1	0.0	0.0	0.0	0.0	0.1
26.20	1513	$\gamma$-Cadinene	Sesquiterpene	C_15_H_24_	39029-41-9	39.0	66.6	12.2	29.4	77.6
26.30	1522	$\delta$-Cadinene	Sesquiterpene	C_15_H_24_	483-76-1	2.1	1.7	0.6	2.0	1.3
26.39	1529	*cis*-Calamenene	Sesquiterpenoid	C_15_H_22_	72937-55-4	26.2	12.2	15.7	29.0	3.1
26.54	1533	*trans*-Cadina-1,4-diene	Sesquiterpene	C_15_H_24_	38758-02-0	6.5	3.3	3.7	6.8	0.6
26.77	1537	$\alpha$-Cadinene	Sesquiterpene	C_15_H_24_	82468-90-4	13.0	11.1	6.6	13.9	3.9
26.90	1544	$\alpha$-Calacorene	Sesquiterpenoid	C_15_H_20_	21391-99-1	1.8	1.4	0.9	1.8	0.3
27.80	1554	$\beta$-Vetivenene	Sesquiterpenoid	C_15_H_22_	27840-40-0	0.0	0.0	2.7	4.3	0.2
30.02	1675	Cadalene	Sesquiterpenoid	C_15_H_18_	483-78-3	11.5	2.7	10.8	12.5	0.6

### *P. microcarpus* displays a repression of four TPS-encoding genes during host colonization

**Fig. 2:**
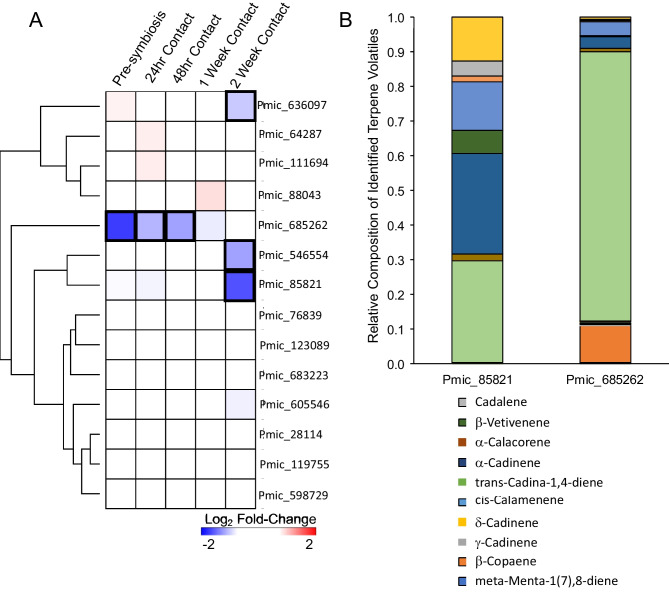
*P. microcarpus* terpene synthase genes are repressed during colonization by *E. grandis* and catalyze the synthesis of sesquiterpenes*.* **A** Differential expression of *P. microcarpus* genes annotated as terpene synthases during colonization of *E. grandis.* All values are Log2 fold change values of expression in fungi colonizing the host compared to axenically grown fungal colonies. The results were hierarchically clustered using Euclidean distance. Bold boxes around a specific value indicate significant DEG (≥ 2× fold change, *P* < 0.05). **B** Volatile metabolites produced by liquid cultures of *Escherichia coli* Rosetta2 (DE3) pLysS heterologously expressing either *P. microcarpus* protein Pmic_685262 or Pmic_85821. The headspace above the induced cultures was sampled by SPME and analyzed by GC-MS. The identified sesquiterpene compounds that were not found in *E. coli* Rosetta2 (DE3) pLysS cultures containing an empty expression vector are reported here as percentages of the total identified sesquiterpenes using semi-quantitative analysis

Because little is known about the TPS genes of *P. microcarpus*, we sought to better characterize the terpene synthases of this fungus. Surprisingly, in contrast to the *L. bicolor* model (Martin et al. 2008), we found that the majority of TPS-encoding genes were not regulated by the presence of the plant host *E. grandis* (Fig. 2A; Supplemental Table 1)*.* Only four genes were significantly regulated during colonization, all of which were repressed (*Pmic_636097, Pmic_685262, Pmic_546554, and Pmic_85821*; FC ≤ -2x; p < 0.05). Genes coding for the *P. microcarpus* TPS proteins Pmic_636097, Pmic_546554, and Pmic_85821 were significantly downregulated during the functional symbiotic stage of colonization (i.e., 2 weeks post-contact, when the fungus is fully established in the root and nutrients are exchanged between the two partners according to Plett et al. 2020). Meanwhile, the *P. microcarpus* gene encoding protein Pmic_685262 was largely repressed during the early stages of pre-symbiosis and mantle formation, after which it resumed normal levels of expression (Fig. 2A).

Two of the four differentially regulated TPS genes belonged to the poorly studied clade V (*Pmic_85821* and *Pmic_685262*; Fig. 1A). Despite being in the same clade, they only exhibit 21.5% amino acid sequence similarity. The volatile profiles were nearly identical to those of *L. bicolor* TPS genes (Fig. 2B). Pmic_85821 produced two terpenoids in roughly equal quantities: $\gamma$-cadinene (29.4%) and *cis*-calamenene (29%). This synthase also led to a high proportion of $\alpha$-cadinene (13.9%) and cadalene (12.5%; Table 1). Pmic_685262, meanwhile, produced predominantly $\gamma$-cadinene (77.6%), followed by 10.6% meta-mentha-1(7),8-diene (Table 1).

### Symbiosis between *E. grandis *and *P. microcarpus* results in the release of a complex mixture of volatile terpenes

**Fig. 3:**
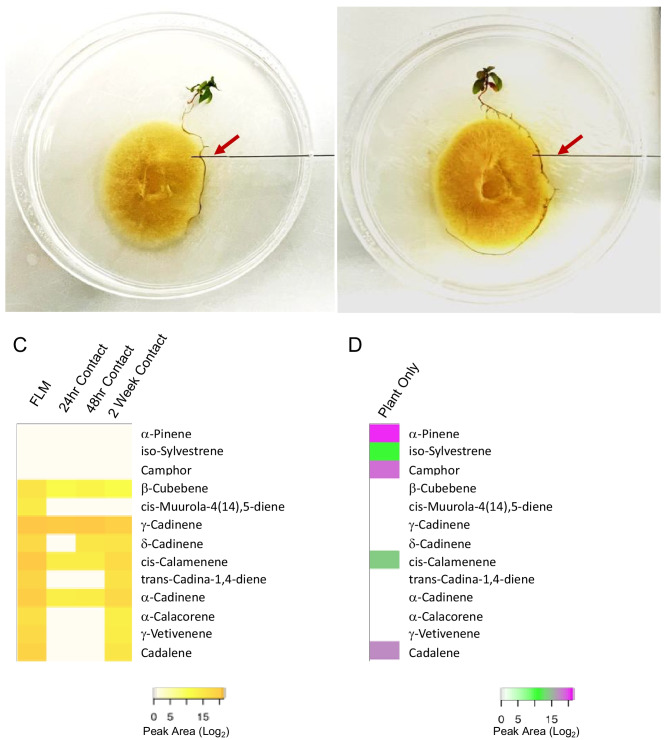
Volatile di- and sesquiterpenes are repressed during the early stages of colonization. **A** Microcosm with *P. microcarpus* and an *E. grandis* seedling 24 h post-contact showing headspace sampling with an SPME fiber (arrow). **B** Microcosm with *P. microcarpus* and an *E. grandis* seedling 2 weeks post-contact showing headspace sampling with an SPME fiber (arrow). **C** Scaled peak area (Log2) of mono-, di-, and sesquiterpene volatiles identified in the headspace of axenically grown free-living mycelia of *P. microcarpus* (FLM) and at 24 h, 48 h, and 2 weeks after contact with the roots of an *E. grandis* seedling. **D** Scaled peak area (Log2) of mono-, di-, and sesquiterpene volatiles identified in the headspace of axenically grown *E. grandis* seedlings

Given that four *P. microcarpus* TPS-encoding genes were suppressed during the different stages of host colonization, we hypothesized that the detectable terpene mixtures evolved during the same time frame would be less complex than the growth of the two organisms individually. Using SPME fibers, we measured the volatile profiles above free-living mycelium (FLM) of axenically grown *P. microcarpus,* in axenically grown *E. grandis* seedlings (i.e., plant only), and at three stages post-contact between the fungus and the plant root (Fig. 3A, B). In FLM, we detected very high levels of ten separate terpenes that were dominated by sesquiterpenes (Fig. 3C). Interestingly, a much less complex mixture was detected above the roots of *E. grandis* when grown axenically, with only three terpenes identified, dominated by monoterpenoids (e.g., pinene; Fig. 3D). The relative level of detectable terpenes dropped precipitously from 24 h post-contact through 2 weeks of colonization as compared to either FLM or plant only controls (Fig. 3C). $\gamma$-Cadinene, for example, was the most highly detected terpene in FLM (39% of all volatile terpenes detected) but dropped to nearly half of its original levels 24 h post contact with the host and then down to 24% of its original concentration following 2 weeks of contact. It was also found that *cis*-calamenene was one of the most highly detectable volatile terpenoids in FLM (25.8% of all terpene volatiles), a level that dropped by 100 × following contact with the host. These two compounds were found to be products of recombinant Pmic_85821 and Pmic_685262 (Fig. 2B). Therefore, while it should be noted that this latter compound, along with cadalene, cannot be assigned specifically to either the fungus or the plant during colonization as both organisms produce the same volatile, their production profile is commensurate with the decrease in gene expression in *P. microcarpus* (Fig. 2A).

### *E. grandis* terpene synthases are not expressed in roots during symbiosis

**Fig. 4:**
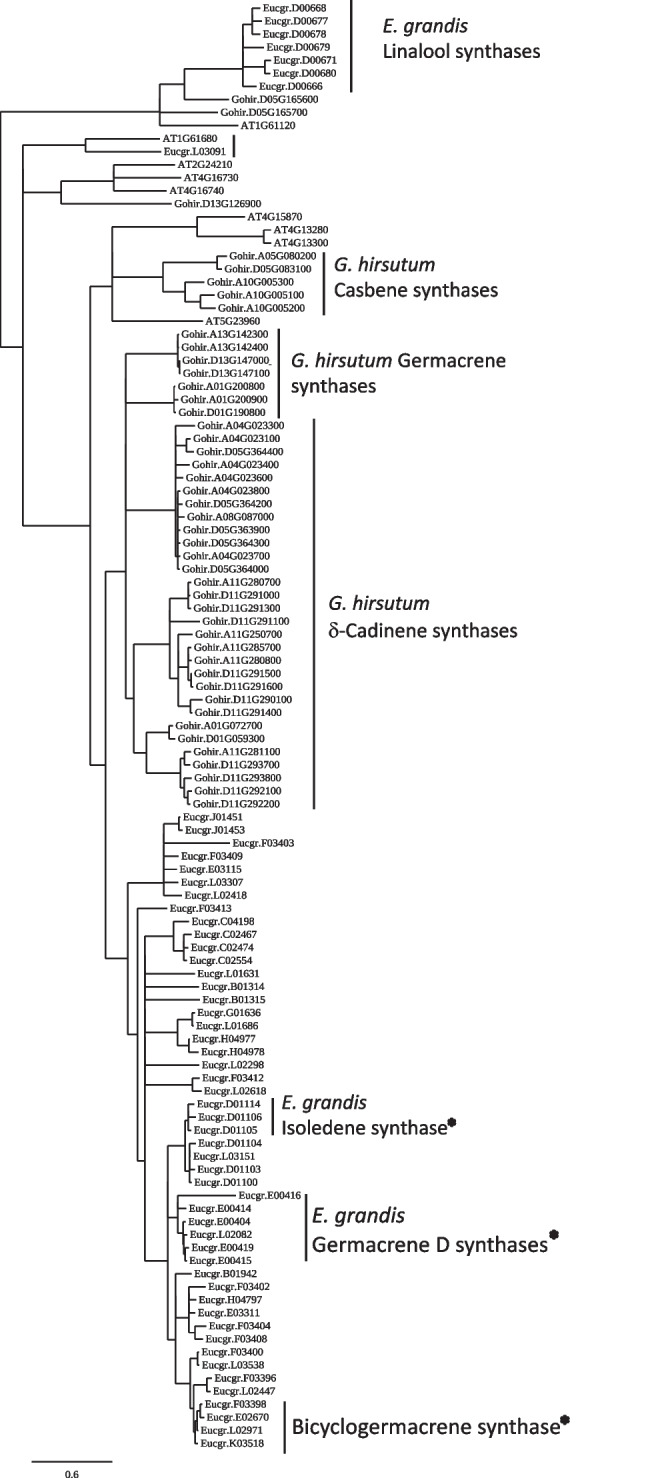
Unrooted maximum likelihood phylogram of TPS homologs identified in the three-plant model systems. * indicates activity based on functional proof of one or more proteins based on the study by Külheim et al. (2015). The remaining assigned activities were based on the Phytozome annotations (accessed April 2023) for *E. grandis* and *A. thaliana.* Annotations for *G. hirsutum* were based on annotations from Chen et al. (2020a, b)

*E. grandis* encodes 114 predicted mono- and di-terpene synthases (Myburg et al. 2014; Külheim et al. 2015), very few of which have been functionally characterized. To gain a better understanding of the clades of terpene synthases that may be expressed in the roots of *E. grandis,* we compared the protein sequence of *E. grandis* terpene synthases to the recently published cotton genome (Chen et al. 2020a, b), a plant known to produce a variety of terpenes. We also included sequences from *A. thaliana* (Fig. 4). With the exception of seven predicted linalool synthases and one putative nerolidol synthase, all *E. grandis* terpene synthase genes were grouped separately from the two other plant models. We next investigated the expression of *E. grandis* genes in the roots of axenically grown seedlings and during the colonization process. Only one gene, *Eucgr.F03855*, a predicted Ent-Kaur-16-Ene synthase, had detectable levels of expression in the roots as per our parameters given in the “Methods” section (Supplemental Table 2). This expression did not change significantly across any of the time points (*P* > 0.05).

### *P. microcarpus* terpenes alter root growth and colonization potential

**Fig. 5:**
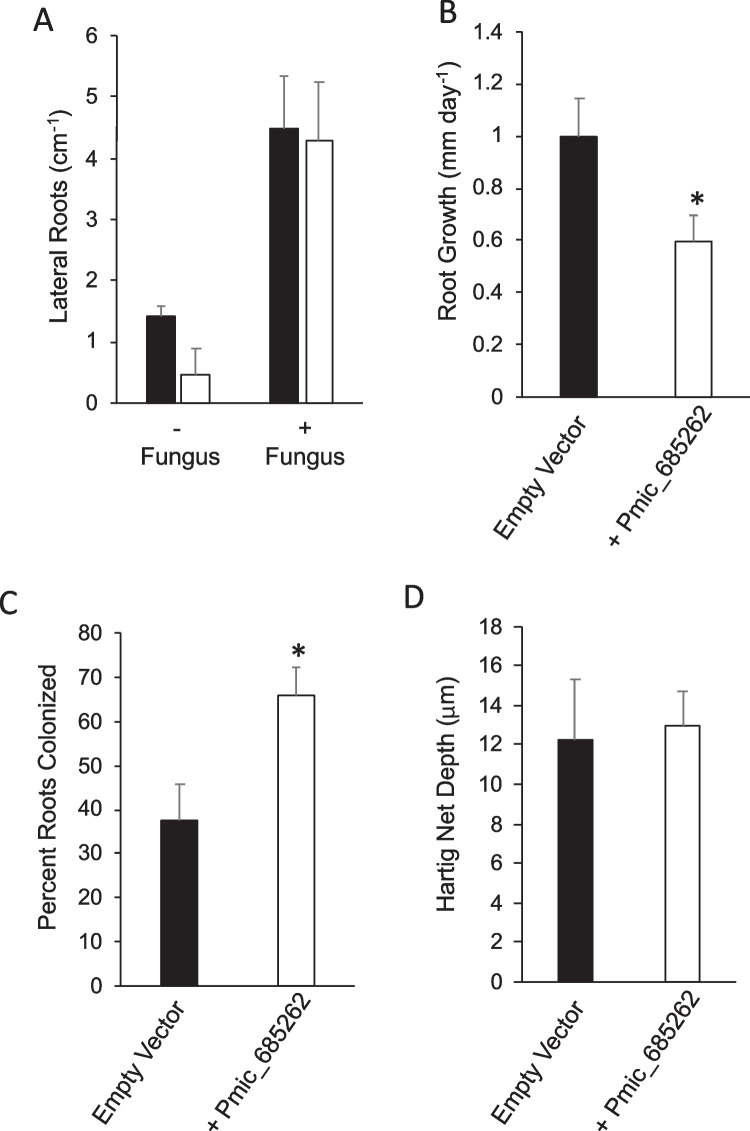
Increased levels of $\gamma$-cadinene reduces *E. grandis* root growth and increases *P. microcarpus* root colonization. **A** Lateral roots per centimeter of *E. grandis* root after 2 weeks of growth in a microcosm with *E. coli* expressing either an empty vector (black bars) or Pmic_685262 (white bars). Lateral root induction was counted either in the absence of *P. microcarpus* (− fungus) or when in contact with *P. microcarpus* (+ fungus). **B** Root growth rate of *E. grandis* seedlings averaged over 2 weeks of growth in a microcosm with *E. coli* expressing either an empty vector (black bars) or Pmic_685262 (white bars). **C** Percentage of *E. grandis* lateral roots exhibiting colonization by *P. microcarpus* (determined by the presence of mantle and arrested root growth) in a microcosm with *E. coli* expressing either an empty vector (black bars) or Pmic_685262 (white bars). **D** Degree of *P. microcarpus* hyphal ingrowth into the apoplastic space of colonized roots of *E. grandis* (i.e., Hartig net depth) in a microcosm with *E. coli* expressing either an empty vector (black bars) or Pmic_685262 (white bars). All bar charts ± standard error; * denotes significant difference in Pmic_685262 treatment from the empty vector (*P* < 0.05; two-tailed Student’s t-test) (*n* = 15)

Given the role of terpenes in plant defense, the finding that *P. microcarpus* produces a wide variety of terpenes has led to the question of their role in colonization. As one of the major products of all terpene synthases tested was $\gamma$-cadinene, we focused on the role of this compound. As we were unable to source a pure standard of this volatile, we utilized *E. coli* producing Pmic_685262, as it had the simplest volatile profile of all the enzymes (Fig. 2B). Using this heterologously produced terpene, we supplemented the level of terpenes in the headspace of microcosms with either axenically grown *E. grandis* seedlings or *E. grandis* seedlings undergoing colonization by *P. microcarpus*. Lateral root development per centimeter was reduced in *E. grandis* seedlings exposed to elevated terpenes, but this was not significant compared to the control (*P* = 0.16; Fig. 5A). When fungus was present, there was no difference in lateral root development between the terpene addition and control groups (Fig. 5A). Terpene enrichment led to a significant reduction in root extension (Fig. 5B) and a significant increase in the percentage of lateral roots colonized by *P. microcarpus* (defined by the presence of a mantle; Fig. 5C). Fungal ingrowth to form the Hartig net was unaffected by terpene addition (Fig. 5D).

## Discussion

Compared with other fungal lifestyles, several ECM fungi appear to be especially enriched in terpene synthases (Lofgren et al. 2021). However, given the breadth of TPSs encoded across the fungal tree of life, the products catalyzed by these proteins are very difficult to predict. Phylogeny combined with selected enzyme characterization has been used in saprotrophic fungi to assign functional clades based on reaction mechanisms (Agger et al. 2009; Lin et al. 2017; Wawrzyn et al. 2012; Wu et al. 2022). Four to six terpene synthase clades have been previously defined, depending on the breadth of the fungal genomes utilized (Chen et al. 2021; Wu et al. 2022). Clades I–IV have enzymes from saprotrophic fungi that have been characterized, but little is known about the activity of clade V (Agger et al. 2009; Lopez-Gallego et al. 2010; Wawrzyn et al. 2012). The majority of studies focusing on identifying and characterizing volatile terpenes in ECM fungi have looked at the headspace above fungal hyphae (Abdulsalam et al. 2021; Ditengou et al. 2015; Ezediokpu et al. 2022). More recently, one study was published using an ECM fungus where the authors heterologously expressed individual terpene synthases to determine their soluble products (Nosenko et al. 2023). Phylogenetically, the two fungal models considered here had protein orthologs that fell across the majority of the five major clades. Two of the functionally tested *L. bicolor* proteins from clade II, selected based on their induction during symbiosis with a host plant (Martin et al. 2008), produced primarily $\gamma$-cadinene with *cis*-calamenene as a secondary product, whereas the third protein from this clade primarily produced linalool. Differences in primary terpene products have been noted in previous studies despite their close phylogenetic similarity. For instance, Cop1 and Cop2 from *Coprinopsis cinerea* and Omp1 of *Omphalotus olearius* are both from the same clade I branch of terpene synthases, yet the former two produce germacrene, while the latter produces primarily $\alpha$-muurolene (Agger et al. 2009; Wawrzyn et al. 2012). In clade II, Omp 5a/b and Omp4 produce $\beta$-elemene and $\delta$-cadinene, respectively, whereas two of the synthases from *L. bicolor* characterized here from this clade produced $\gamma$-cadinene and linalool (Wawrzyn et al. 2012; Fig. 1). We also sought to extend our knowledge of clade V synthases and found that one of the enzymes (Pmic_685262) produced primarily $\gamma$-cadinene with traces of other compounds, while the other protein (Pmic_85821) produced a more complex mixture of $\gamma$-cadinene, *cis*-calamenene, $\alpha$-cadinene, and cadalene. The similarity between clade II and clade V terpene products was unexpected given the apparent phylogenetic divergence but may make sense given the differences in terpene products, even within a clade of terpene synthase enzymes. Therefore, while phylogenetic similarity of terpene synthases in fungi may be useful in providing this gene family with a clade structure, it does not appear to properly predict the differences in their actual biochemical products, indicating a need for more in-depth functional characterization of these enzymes across the fungal tree of life.

Given the well-studied roles of plant terpenes, including the cadinene class of sesquiterpenes which act as defense primers against fungal invasion (Bianchini et al. 1999; Liu et al. 2021; Townsend et al. 2005), it seems counterintuitive that mutualistic symbiotic fungi express similar synthases during host colonization. However, previous evidence from *L. bicolor* and *T. vaccinum* clearly demonstrates that the expression of genes associated with terpene synthases is induced during the early stages of interaction with a receptive plant host (Martin et al. 2008; Ezediokpu et al. 2022). In addition, in the headspace of both fungi, researchers have detected higher levels of volatile terpenes during colonization (Abdulsalam et al. 2021; Ditengou et al. 2015; Ezediokpu et al. 2022). In the case of *T. vaccinum,* these terpenes can be elevated for weeks following contact between the fungus and host root, suggesting that they are not inhibitory to symbiosis (Ezediokpu et al. 2022). Contrary to our hypothesis, however, the *P. microcarpus* system behaved differently. While we were able to detect strong gene expression of many terpene synthases at the transcript level and we identified a rich mixture of volatile terpenes in the headspace above axenically grown mycelia, the expression of these genes was either unaffected by the presence of the host or, in the case of four genes, repressed at different points of colonization. Commensurate with the gene expression findings, we also found that many of the terpenes found produced by free-living mycelium disappeared within 24 h of fungal contact with a host root or, as in the case of $\gamma$-cadinene, decreased steadily across colonization. While it is possible that these findings could be influenced by the fact that the microcosm construction used is not soil-based, experiments with *L. bicolor:Populus* (Ditengou et al. 2015) and with *T. vaccinum:Picea abies* (Abdulsalam et al. 2021) were also performed with a similar soilless system. Therefore, our results suggest that volatile terpenes in *P. microcarpus* may play an early role prior to plant contact, a role that is attenuated during the initial stages of host colonization and integration.

Several alterations in the plant root system occur during the initiation of symbiotic interactions with ECM fungi (Felten et al. 2009). These include an increase in lateral rooting, a slowing and eventual cessation of root growth, and finally loosening of cell wall connections between plant epidermal cells, enabling ingrowth of fungal hyphae into the root apoplast (Garcia et al. 2015; Martin et al. 2017). While the latter stages are mediated in part by cell wall-modifying enzymes released from the fungus (Casarrubia et al. 2018; Zhang et al. 2018), evidence suggests that volatile terpenes induce lateral rooting in plant roots before fungal contact (Ditengou et al. 2015). Contrary to our hypothesis, exposure of *E. grandis* to one of the most prevalent volatiles from *P. microcarpus* (i.e., $\gamma$-cadinene) did not increase lateral rooting in either the absence or presence of the fungus. However, we were correct in hypothesizing that the increase in $\gamma$-cadinene would lead to a significant increase in the number of colonized lateral roots. Our results would suggest that this effect is due in part to $\gamma$-cadinene reducing the rate of root extension. Previous studies have shown that rapid root growth, in part due to the loss of signals that control this aspect during ECM colonization, results in aborted mycorrhizal root tips (Clowes 1981; Tam and Griffiths 1994; Wong-Bajracharya et al. 2022). Therefore, by slowing root growth, $\gamma$-cadinene would allow for a greater length of time for the fungus to form a mantle around the root and release further diffusible signals (e.g., proteins and miRNA) that enable a stably colonized root tip. In a soil system, this could slow root growth to a point where the fungus is able to grow up to the root and begin mantle formation. This role aligns well with the expression pattern of these terpene synthases, which are highly expressed until fungal contact with the root.

Sampling of volatile terpenoids in microcosms with both an ECM fungus and its host leads to the possibility that plant terpenoids are captured and quantified in addition to fungal secondary metabolites. Myrtaceae, which includes *E. grandis*, produces many different terpenoids that are involved in a range of physiological and symbiotic processes (Hill et al. 2022; Padovan et al. 2014; Voelker et al. 2023). While these secondary metabolites have been implicated in defense against pathogenic organisms (Yong et al. 2019; Hsieh et al. 2021; Moffitt et al. 2022), little is known about their role in mutualistic symbiosis with ECM fungi. In other plant-fungal interactions, it is possible for the fungus to alter or repress host terpenoid production. For example, Nones et al. (2022) reported that fungal propagation in stem tissues by beetles led to reduced plant monoterpenes. We saw a similar impact during *P. microcarpus* colonization of *E. grandis* roots: prior to symbiosis, the roots produced three main monoterpenes ($\alpha$-pinene, iso-sylvestrene, camphor), monoterpenes that were undetectable following contact with *P. microcarpus.* We also found no discernible expression of terpene synthases in *E. grandis* roots following fungal contact. While we do not know the mechanism of this repression, nor was there a mechanism proposed by Nones et al. (2022), a recent study on the *interaction of Populus* with *L. bicolor* suggests that fungal-secreted proteins may control plant terpene production (Marques-Galvez et al. 2022). Plant-interacting fungi release a suite of small secreted proteins that alter plant physiology and promote symbiosis (Plett and Plett 2022; Snelders et al. 2022). One of these, LbMiSSP7, produced by *L. bicolor,* interacts with a host jasmonic acid (JA) co-receptor, which represses JA-induced gene transcription (Daguerre et al. 2020; Plett et al. 2014). Marques-Galvez and colleagues (2022) found that these genes were enriched in terpene synthases and that by interrupting their expression, host terpene production decreases during colonization between *L. bicolor* and *Populus.* Therefore, it is possible that a similar mechanism is at play in the *P. microcarpus:E. grandis* system to explain the repression of host terpene synthases, although at present, it is unknown what fungal proteins may play a similar role to LbMiSSP7.

Altogether, our work demonstrates the co-evolutionary similarity of the role of volatile terpenes in distantly related ECM fungi. Our results suggest that fungal terpenes may improve fungal colonization success by slowing root growth prior to fungal contact, an action that likely improves mantle formation. These results are very interesting as they highlight the possible divergent roles of terpenoid compounds produced by fungi and plants, whereby the former are induced during specific phases of the interaction and the latter are repressed for successful integration of the fungus into the root. In the larger ecological context concerning the role of terpenoids in plant-microbe interactions, our results further highlight the importance of volatile secondary metabolites as cross-kingdom signals necessary for the coordinated development of the two organisms during mutualistic symbiosis.

### Supplementary Information

Below is the link to the electronic supplementary material.

Supplementary file1 (XLSX 11 KB)

Supplementary file2 (XLSX 17 KB)
